# Nanoscale ductile fracture and associated atomistic mechanisms in a body-centered cubic refractory metal

**DOI:** 10.1038/s41467-023-41090-3

**Published:** 2023-09-08

**Authors:** Yan Lu, Yongchao Chen, Yongpan Zeng, Yin Zhang, Deli Kong, Xueqiao Li, Ting Zhu, Xiaoyan Li, Shengcheng Mao, Ze Zhang, Lihua Wang, Xiaodong Han

**Affiliations:** 1 Beijing Key Lab and Institute of Microstructure and Properties of Advanced Materials, Beijing University of Technology, 100124 Beijing, China; 2 CAS Key Laboratory of Mechanical Behavior and Design of Materials, Department of Modern Mechanics, University of Science and Technology of China, 230026 Hefei, China; 3 Woodruff School of Mechanical Engineering, Georgia Institute of Technology, Atlanta, GA 30332 USA; 4 Centre of Advanced Mechanics and Materials, Applied Mechanics Laboratory, Department of Engineering Mechanics, Tsinghua University, 100084 Beijing, China; 5 State Key Laboratory of Silicon Materials, Zhejiang University, 310008 Hangzhou, China

**Keywords:** Condensed-matter physics, Mechanical engineering, Metals and alloys

## Abstract

Understanding the competing modes of brittle versus ductile fracture is critical for preventing the failure of body-centered cubic (BCC) refractory metals. Despite decades of intensive investigations, the nanoscale fracture processes and associated atomistic mechanisms in BCC metals remain elusive due to insufficient atomic-scale experimental evidence. Here, we perform in situ atomic-resolution observations of nanoscale fracture in single crystals of BCC Mo. The crack growth process involves the nucleation, motion, and interaction of dislocations on multiple 1/2 < 111 > {110} slip systems at the crack tip. These dislocation activities give rise to an alternating sequence of crack-tip plastic shearing, resulting in crack blunting, and local separation normal to the crack plane, leading to crack extension and sharpening. Atomistic simulations reveal the effects of temperature and strain rate on these alternating processes of crack growth, providing insights into the dislocation-mediated mechanisms of the ductile to brittle transition in BCC refractory metals.

## Abstract (web summary)

Understanding the competing modes of brittle versus ductile fracture is critical for preventing material failure. Here, the authors reveal the atomic-scale processes of crack growth in Mo crystals, providing mechanistic insights into the ductile to brittle transition in BCC refractory metals.

## Introduction

Body-centered cubic (BCC) refractory metals with high melting temperature usually exhibit high strength but suffer from low tensile ductility and fracture toughness^1–4^. Such conflicts between strength, ductility, and toughness can severely limit practical applications of BCC-refractory metals. To overcome these conflicts, it is important to understand the competing modes of brittle and ductile fracture in the BCC-refractory metals^5–11^. Rice and Thomson^12,13^ developed a classical theory of crack-tip dislocation nucleation in a dislocation-free crystal, which determines whether a crack can propagate in a fully brittle mode. According to that theory, BCC-refractory metals would exhibit brittle fracture through cleavage crack propagation without dislocation activity. Many experimental, theoretical, and computational studies^14–25^ have investigated the crack-tip dislocation and fracture processes to enhance the fundamental understanding of ductile and brittle cracking. In particular, transmission electron microscopy (TEM) studies have reported observations of crack-tip dislocations^14–16^, and atomistic simulations have revealed the nucleation and motion of crack-tip dislocations^18–25^. However, the role of the dynamic interplay between crack-tip dislocation processes and crack growth behavior remains unclear due to insufficient atomic-scale experimental evidence. Hence, uncertainties remain regarding the microscopic mechanisms of the ductile-to-brittle transition in BCC-refractory metals.

Recent advances in the in situ high-resolution TEM (HRTEM) experimental techniques^26–29^ have offered opportunities to understand the fundamental mechanisms of crack formation and propagation in BCC-refractory metals. So far, these in situ HRTEM techniques have not yet been applied to investigate the crack-tip processes in BCC-refractory metals. Most of the previous in situ TEM studies were performed with relatively low-resolution imaging, which does not allow for a detailed characterization of atomic-scale crack-blunting and sharpening processes. Consequently, our understanding of these phenomena heavily relies on molecular dynamics (MD) simulations. Nonetheless, in situ atomic-resolution experimental investigations are essential for a deeper understanding of the crack growth mechanisms and underlying dislocation activities in BCC metals, and they are still lacking to date.

In this work, we conduct in situ atomic-resolution observations of nanoscale fracture in single crystals of BCC Mo. We find that the crack growth process involves the nucleation, motion, and interaction of dislocations at the crack tip. These dislocation activities give rise to a competition between the alternating processes of crack-tip plastic shearing and local separation normal to the crack plane. The former process results in crack blunting and local thinning, while the latter leads to crack extension and sharpening. Such competition involves more complex dislocation processes than the competition between crack-tip dislocation emission and cleavage fracture in a dislocation-free crystal studied in previous works^12^. In addition, our MD simulations demonstrate the effects of temperature and strain rate on the two competing processes that govern the ductile-to-brittle transition. Hence, the combined experimental and MD results provide an in-depth understanding of the atomic-scale mechanisms of the ductile-to-brittle transition in BCC-refractory metals.

## Results

### In situ HRTEM imaging

**Fig. 1:**
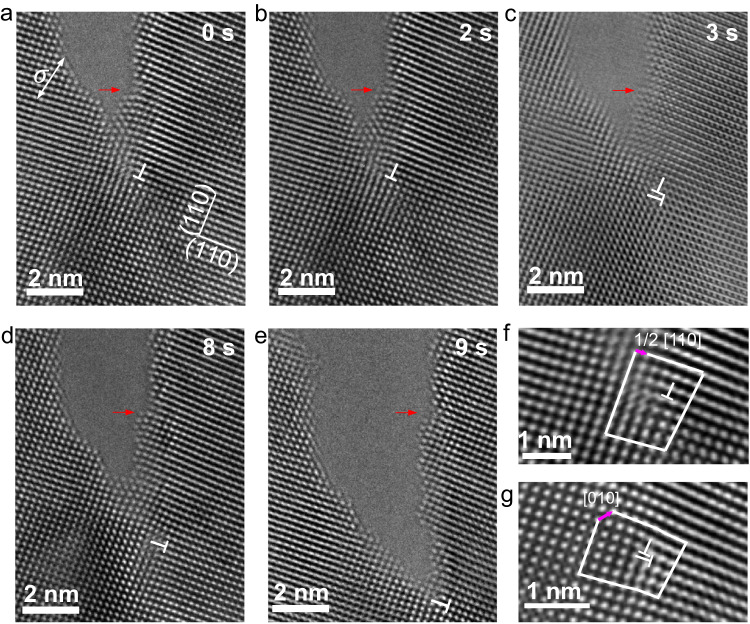
HRTEM observations of the blunting and growth of a ($5\bar{1}0$)[010] crack due to crack-tip dislocation emission. The white arrow indicates the tensile loading direction, while the red arrow in all HRTEM images marks the initial crack-tip location at the beginning of in situ testing (*t* = 0 s). **a**, **b** Crack-tip nucleation of a full dislocation with a 3D Burgers vector **b** = 1/2[111]($1\bar{1}0$) or 1/2[$11\bar{1}$]($1\bar{1}0$). **c** Formation of a dislocation lock with **b** = [010](100) from two closely spaced dislocations lying on the (110) and ($1\bar{1}0$) planes, respectively. **d** Crack growth. The current crack-tip position is ~2 nm away from the initial crack-tip location, as indicated by the reference red arrow. A dislocation on the (110) plane is several atomic layers ahead of the current position of the crack tip. **e** Further crack growth. A dislocation on the (110) plane is close to the current position of the crack tip. **f** Burgers loop analysis of a single dislocation. It indicates the projected in-plane Burgers vector 1/2[110]($1\bar{1}0$) of this single dislocation. **g** Burgers loop analysis of a dislocation lock, with the Burgers vector **b** = [010](100) as indicated by the pink arrow.

Figure 1 displays a time series of in situ HRTEM images showing crack growth in a Mo nanocrystal, observed along the [001] zone axis. These images were acquired at a rate of two frames per second. A pre-existing crack formed on the ($5\bar{1}0$) plane. In Fig. 1a, the white arrow indicates the tensile loading direction, while the red arrow marks the initial position of the crack tip at the beginning of in situ testing (*t* = 0 s). The red arrow is displayed in all subsequent HRTEM images (Fig. 1b–e) to track the distance between the initial and current position of the crack tip. In Fig. 1a, a dislocation appeared in front of the crack tip, and it is marked by ⊥ at the termination of the extra half (110) plane at the dislocation core. Burgers circuit analysis of an enlarged HRTEM image (Fig. 1f) revealed that the in-plane Burgers vector of this dislocation is 1/2[110]($1\bar{1}0$), which is the projection of the three-dimensional (3D) Burgers vector **b** = 1/2[111]($1\bar{1}0$) or 1/2[$11\bar{1}$]($1\bar{1}0$) in the BCC lattice. This type of crack-tip structure persisted for ~2 s (Fig. 1b). Subsequently, another dislocation nucleated (Fig. 1c) with an in-plane Burgers vector 1/2[$\bar{1}10$](110), which is the projection of the 3D Burgers vector **b** = 1/2[$\bar{1}11$](110) or 1/2[$\bar{1}1\bar{1}$](110). This dislocation considerably reduced lattice distortion near the crack tip, which can be attributed to local stress relaxation resulting from the emission and motion of crack-tip dislocations. Figure 1d shows that the current position of the crack tip was approximately 2 nm from its initial location, as indicated by the red arrow. The crack extension was accompanied by crack blunting, caused by the emission and motion of crack-tip dislocations. Whereas only a single dislocation with a projected in-plane Burgers vector 1/2[$\bar{1}10$](110) was captured near the crack tip in Fig. 1d, numerous dislocations were emitted continuously at the crack tip during the loading process, leading to propagation of the crack tip (see Supplementary Movies 1 and 2). With increasing load, the crack continued to grow, as shown in Fig. 1e where a crack-tip dislocation with a projected in-plane Burgers vector 1/2[$\bar{1}10$](110) was observed. Overall, our in situ atomic-scale observations revealed that nanoscale crack growth in BCC Mo involves crack-tip dislocation emission and motion rather than brittle cleavage fracture.

Crack-tip dislocations can react with each other to form dislocation locks. Figure 1g shows two closely spaced dislocations with respective extra half-planes of (110) and ($1\bar{1}0$). The overlapping core structures indicate the formation of a dislocation lock with a net Burgers vector **b** = [010](100), as shown by the Burgers loop in Fig. 1g; other examples of dislocation locks are shown in Supplementary Figs. 1 and 2. The reaction of lock formation can be expressed as 1/2[$\bar{1}11$](110) + 1/2[$11\bar{1}$]($1\bar{1}0$) → [010](100) or 1/2[$\bar{1}1\bar{1}$](110) + 1/2[111]($1\bar{1}0$) → [010](100). This type of overlapping core structure could correspond to the projection of atom columns in the core region of a 3D “Y”-shaped dislocation lock (Supplementary Fig. 3). These dislocation locks are stable junctions that can not only trap the involved dislocations but also impede the motion of neighboring dislocations. The resulting dislocation accumulation increased the resistance to crack growth. However, the increased load can cause the destruction of dislocation locks^30^, enabling further dislocation emission from the crack tip.

**Fig. 2:**
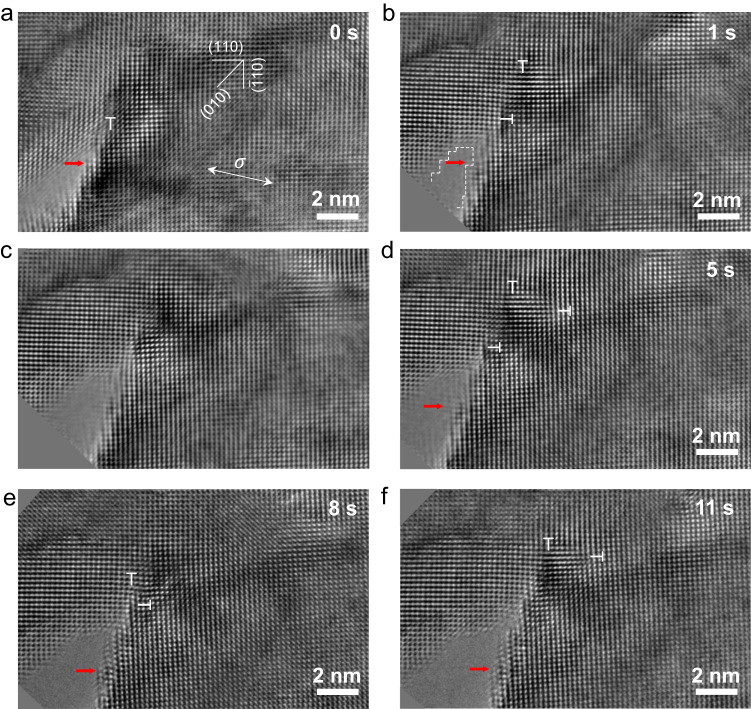
HRTEM observations of the blunting and growth of a (010)[001] crack due to crack-tip dislocation emission. The white arrow indicates the direction of tensile load and the red arrow in all HRTEM images marks the initial crack-tip location at the beginning of in situ testing (*t* = 0 s). **a** Crack-tip nucleation of a full dislocation with a 3D Burgers vector **b** = 1/2[$\bar{1}11$](110) or 1/2 [$\bar{1}1\bar{1}$](110). The distance between the dislocation and the crack tip is four atomic layers. **b** The nucleated dislocation moves away from the crack tip, followed by the nucleation of a new dislocation with **b** = 1/2[111]($\bar{1}10$) or 1/2[$11\bar{1}$]($\bar{1}10$) at the crack tip. **c**–**f** Crack blunting and growth, resulting from subsequent crack-tip dislocation emission events under increasing load. The images in (**c**–**e**) show that crack blunting occurs by the generation of (110) and $(\bar{1}10)$ crack edges at an angle of ± 45° with respect to the (010) crack plane. The images in (**e**, **f**) show that further crack extension occurs by local separation normal to the (010) crack plane, evident by the narrow lattice layer with light contrast (implying reduced sample thickness) ahead of the crack tip.

Figure 2 shows another example of in situ HRTEM observation of crack growth, as viewed along the [001] zone axis. In this case, a pre-existing crack formed on the (010) plane and exhibited similar dislocation activities and resultant crack extension as shown earlier in Fig. 1. In particular, we observed two alternating processes during crack growth. In Fig. 2a, a crack-tip dislocation with **b** = 1/2[$\bar{1}11$](110) or 1/2[$\bar{1}1\bar{1}$](110) was emitted and located at a distance of only four atomic layers from the crack tip. After 1 s (Fig. 2b), this dislocation moved away from the crack tip, and another dislocation with **b** = 1/2[111]($\bar{1}10$) or 1/2[$11\bar{1}$]($\bar{1}10$) was emitted. Such multiple dislocation emission events at the crack tip resulted in a crack-blunting process, as shown in Fig. 2c–e. Supplementary Movies 1 and 2 show more detailed processes of emission of multiple dislocations from the crack tip and the resultant crack blunting and extension. As a result, the crack tip in Fig. 2 became blunted due to the production of (110) and $(1\bar{1}0)$ crack edges at ± 45° with respect to the overall (010) crack plane. This occurred because the emission of 1/2[111]($\bar{1}10$) and 1/2[$\bar{1}11$](110) dislocations from the crack tip led to slide-offset along the (110) and ($1\bar{1}0$) slip planes (marked with white dashed lines in Fig. 2b). Following crack blunting, local separation normal to the (010) crack plane occurred ahead of the blunted crack tip, resulting in crack sharpening (Fig. 2e, f). Before the separation, a narrow lattice layer with light contrast appeared in front of the crack tip, indicating a decrease in sample thickness along the out-of-plane direction. Such change in sample thickness indicated that the dislocations emitted from the crack tip led to not only crack blunting but also local thinning along the out-of-plane [010] direction. The local separation at the crack tip led to crack extension and sharpening along the (010) crack plane. The two sequential processes of crack blunting and sharpening alternated as the crack propagated. Similar crack growth processes were frequently observed from in situ HRTEM experiments for different crack orientations. These observations indicate that the alternating processes of crack blunting and local separation normal to the crack plane is a general crack growth mechanism (see more examples in Supplementary Figs. 1 and 2).

### MD simulation

**Fig. 3:**
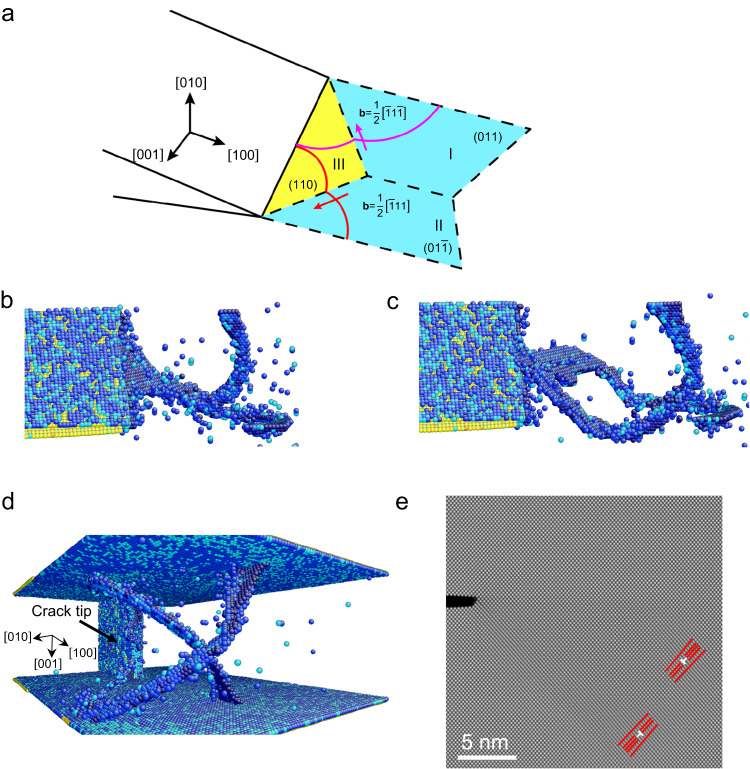
MD simulation results of the nucleation and motion of 3D dislocations at the tip of a (010) [001] crack. **a** Schematic illustration of two 3D-curved dislocation lines nucleated from the crack tip. The two branches of the pink dislocation line are on slip planes I and III and share the common slip direction [$\bar{1}1\bar{1}$]. The two branches of the red dislocation line are on slip planes II and III and share the common slip direction [$\bar{1}11$]. **b**–**d** Sequential MD simulation snapshots showing the nucleation and motion of the two dislocation lines illustrated in (**a**). **e** Simulated HRTEM image using the atomic configuration shown in (**d**). The projected lattice in this image contains dislocations (marked with the symbol ⊥). The red lines indicate the extra half-plane associated with each dislocation. These dislocations have an in-plane Burgers vector 1/2[$\bar{1}10$](110), which corresponds to the 3D Burgers vector **b** = 1/2[$\bar{1}1\bar{1}$](110) and 1/2[$\bar{1}11$](110).

We performed MD simulations to gain a deeper understanding of 3D dislocation processes and the resultant changes in crack structure. Figure 3 shows representative MD results based on an embedded atom method (EAM) potential of Mo^31^ for a (010) crack extending along the [100] direction, which is the same crack orientation as in Fig. 2. Under an increasing tensile load, 3D-curved dislocation lines with the 1/2 < 111 > {110} Burgers vectors nucleated from the crack tip. Due to the high symmetry of the crack and loading orientations, the nucleated dislocations glided on the symmetric {110} slip planes, as illustrated in Fig. 3a. The corresponding MD-simulated images are shown in Fig. 3b–d. Specifically, the pink and red lines in Fig. 3a represent the two curved dislocation lines in Fig. 3b. Each dislocation line nucleated from an intersection between the front of the crack and the side surface of the sample. In addition, each dislocation line has two branches on two intersecting {110} slip planes. For example, the two branches of the pink dislocation line are on slip planes I and III. These two branches share the common slip direction [$\bar{1}1\bar{1}$] parallel to the intersection line between slip planes I and III. Consequently, they can glide on slip planes I and III concertedly. Similarly, the red dislocation line has two branches that share the common slip direction [$\bar{1}11$] and can glide on slip planes II and III concertedly. The activation of these slip systems can be attributed to their similarly large Schmid factors because of the symmetric crack and loading orientations. Incidentally, the concerted gliding of different branches of a 3D dislocation line on intersecting slip planes usually operates during prismatic punching of a dislocation loop^32^. Figure 3a, b also shows that each of the red and pink dislocation lines has one of their branches on slip plane III. These two branches can temporarily merge (Fig. 3b) and then separate to continue their respective gliding (Fig. 3c, d). To correlate the MD-simulated 3D dislocation lines with the projected 2D dislocations observed in the HRTEM images, we used the 3D atomic configuration in Fig. 3d to generate a simulated HRTEM image shown in Fig. 3e. The simulated HRTEM image contains dislocations with the in-plane Burgers vector 1/2[$\bar{1}10$](110), which corresponds to the 3D Burgers vector **b** = 1/2[$\bar{1}1\bar{1}$](110) and 1/2[$\bar{1}11$](110). The dislocation structures in the simulated HRTEM image are consistent with those observed in the HRTEM images (Figs. 1 and 2), confirming the correspondence between the MD-simulated 3D-curved dislocation lines and the projected 2D dislocations in the HRTEM images. Hence, the MD simulation results complement the HRTEM observations by revealing 3D processes of dislocation nucleation, motion, and interaction at the crack tip.

**Fig. 4:**
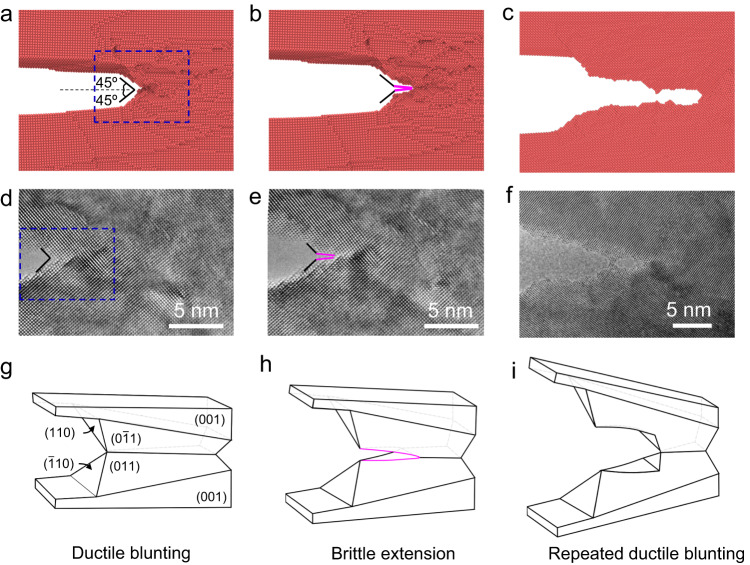
A mixed ductile-brittle mode of crack growth revealed by both MD and HRTEM results of a (010)[001] crack. **a**–**c** MD snapshots showing the alternating processes of ductile blunting and brittle extension of the crack. **a** Ductile blunting of the crack tip occurred due to the formation of a pair of symmetric (110) and ($\bar{1}10$) crack edges (black segments) at an angle of approximately ± 45° with respect to the overall crack plane (dashed line). **b** Crack extension occurred through localized thinning and separation ahead of the crack tip, resulting in horizontal crack edges (pink segments). **c** The alternating processes of crack blunting and extension led to the recurrent horizontal and inclined ( ± 45°) crack edges. **d**–**f** HRTEM images captured the similar crack growth processes as MD snapshots in (**a**–**c**). **g**–**i** Schematic illustrations of the alternating processes of ductile blunting and brittle extension of the crack, corresponding to MD snapshots in (**a**–**c**) and HRTEM images in (**d**–**f**). The schematic in (**g**) represents the boxed region in (**a**) and (**d**).

Furthermore, our MD simulations show that the crack-tip nucleation and motion of 3D dislocation lines can result in crack growth by the alternating processes of crack blunting and local separation. Figure 4a–c shows MD snapshots of a (010) crack, which are consistent with in situ HRTEM observations in Fig. 4d–f (see the corresponding illustration in Fig. 4g–i). The blue dashed boxes in Fig. 4a, d indicate the similar areas in MD simulation and HRTEM image. Starting from an atomically sharp crack tip, crack blunting occurred by the formation of a pair of symmetric (110) and $(\bar{1}10)$ crack edges (marked by the black segments in Fig. 4a and illustrated by the surface facets in Fig. 4g) at ~± 45° with respect to the overall crack plane. Such crack-blunting response is consistent with the in situ HRTEM results in Fig. 2. These two crack edges were generated by crack-tip slide-offset due to dislocation emission along the (110) and ($\bar{1}10$) slip planes. It is worth noting that the formation of two other symmetric crack edges along the (011) and ($0\bar{1}1$) planes (illustrated by the surface facets in Fig. 4g) occurred concurrently in our MD simulations. The resultant dual surface facets were at approximately ± 45° with respect to the TEM image plane and thus not directly visible in the TEM images. Importantly, the dislocations involved in the formation of surface facets have both edge and screw components, resulting in crack blunting as well as local sample thinning ahead of the crack tip. The sample thinning deformation enabled the accommodation of large local stretching along the [010] loading direction. As the applied load increased, the thinning deformation was intensified, leading to the local separation normal to the (010) crack plane. The resulting (010) crack edges (marked by the pink segments in Fig. 4b) are consistent with the in situ HRTEM observations in Fig. 4e, as illustrated in Fig. 4h. The MD and in situ HRTEM results (Fig. 4c, f, respectively) further revealed that crack growth occurred through the alternating processes of crack blunting (resulting in local sample thinning) and local separation normal to the (010) crack plane (leading to crack extension and sharpening), as confirmed by the alternating horizontal and inclined (at ± 45°) crack edges in Fig. 4c, f, i.

## Discussion

In situ TEM observations of crack growth have been reported in the literature^33–35^. However, the insufficient spatial resolution of these studies has made it challenging to correlate the observed crack growth processes with the underlying dislocation activity. In this work, we employed a straining platform capable of achieving atomic-scale resolution to observe the dislocation activity and the resulting changes of crack structure. Consequently, we were able to directly correlate the crack-tip dislocation activity and the atomic-scale crack blunting versus sharpening processes. Modeling the 3D dislocation processes and their resultant effect on crack growth through MD simulations complements HRTEM observations. Our MD simulations indicated that both screw and edge dislocation components became active near the crack tip, where local stresses were high. These results are consistent with previous studies on the enhanced mobility of the screw dislocation component relative to the edge component in BCC nanostructures with prevailing high flow stresses^36^. Hence, the combination of in situ HRTEM observation and MD simulation represents a versatile and effective approach for future investigations of nanoscale fracture in crystalline materials.

In general, the boundary between brittle and ductile fracture is not sharp for many engineering materials. The ductile-to-brittle transition, for example, can involve a competition between plastic shearing (resulting in crack blunting) and local separation normal to the crack plane (leading to crack extension and sharpening)^37,38^. Such competition involves more complex dislocation processes than the competition between crack-tip dislocation emission and cleavage fracture in a dislocation-free crystal^12^. However, the former competition remains elusive due to a lack of direct atomic-scale observations, whereas the latter has been extensively studied to determine whether a crack can propagate in a fully brittle mode. In this work, we provide direct atomic-scale characterization of the former competition, as demonstrated in an HRTEM-scaled Supplementary Movie 1. From this movie, we extract a representative HRTEM image (Supplementary Fig. 4a) which shows a blunted crack tip resulting from plastic shearing and sample thinning. We further extract a subsequent HRTEM image (Supplementary Fig. 4b), which shows a stepwise increase in crack length due to local separation normal to the crack plane. The relative role of the two competing processes in crack growth can be influenced by temperature and strain rate, as shown by MD simulations for a range of temperatures from 10 K to 400 K and a range of strain rates from 1 × 10^7^ to 1 × 10^9 ^s^−1^ (Supplementary Discussion 1 in Supplementary Information). Specifically, increased strain rate and decreased temperature favor more frequent stepwise increases of crack length and less crack blunting, resulting in a more brittle mode of crack growth, and vice versa. Hence, these MD results reveal a transition between crack blunting and crack extension mediated by strain rate and temperature.

Moreover, we performed MD simulations using a modified EAM (MEAM) potential^39^, which can capture the effect of directional bonding in BCC metals. The MEAM-based MD results not only show crack growth through the alternating processes of plastic shearing and local separation, mediated by temperature and strain rate, but also reveal a competing brittle fracture mode of deformation twinning-induced crack extension that predominates at low temperatures and high strain rates (Supplementary Discussion 1). The brittle fracture induced by deformation twinning has been reported by previous experiments on bulk BCC crystals^37,38^. Hence, the MEAM-based MD simulations unveil a broader range of competing processes for the ductile-to-brittle transition in BCC crystals. These MD results provide insights into the effects of temperature and strain rate on the ductile-to-brittle transition, involving the competing processes of plastic shearing, local separation, and deformation twinning during crack growth. Given the frequently observed dislocation activities at the crack tip, it is expected that this mode of ductile-to-brittle transition would occur more commonly in BCC metals, in comparison to the competition between crack-tip dislocation emission and cleavage fracture in a crystal that contains no dislocations. Along this line of research, further in situ HRTEM experiments are needed to gain a deeper understanding of the microscopic mechanisms underlying the ductile-to-brittle transition in the future.

The HRTEM experiments in this study were conducted using thin-foil samples, and the observed fracture processes operated favorably under plane-stress conditions. Therefore, the insights gained could be applied to understand bulk fracture processes in thin samples under plane-stress conditions, assuming that the plane-stress bulk fracture does not involve competition with additional mechanisms such as grain boundary fracture. However, caution should be taken when applying the insights gained to analyze bulk fracture processes in thick samples under plane-strain conditions, as they involve large triaxiality^37,38^. Similarly, supposing no additional competing mechanisms such as grain boundary fracture or growth and coalescence of microcavities are involved, the competition between plastic shearing and local separation normal to the crack plane, as observed in this work, would play an important role in the ductile-to-brittle transition. Such transition is affected by the well-recognized effect of triaxiality^37,38^. That is, the triaxial stress state at a crack tip can increase the effective local yield stress to about three times the far-field tensile yield stress, thereby resulting in a shift of the ductile-to-brittle transition condition during plane-strain fracture relative to plane-stress fracture.

Since our experiments were conducted inside a high vacuum chamber of TEM and the sample was nearly free of impurities, the observed dislocation-crack interactions should reflect their inherent responses in pure Mo single crystals. Previous studies show that impurities such as oxygen and hydrogen prefer to accumulate at the crack tip and grain boundaries, which can increase the energy barriers of crack-tip dislocation emission and glide, thus promoting brittle cleavage^21,40,41^. In an impure polycrystalline microstructure, the competition of crack growth along grain boundaries and inside grains can be influenced by the relative affinities of impurities with the grain boundaries and grain lattice. Such different affinities of impurities can further influence the dislocation-crack interactions at grain boundaries and inside grain interiors, thereby affecting the ductile-to-brittle transition in impure polycrystalline microstructures. Hence, our in situ HRTEM study lays the groundwork to further investigate dislocation-crack interactions in impure polycrystalline microstructures, toward a deeper understanding of impurity effects on ductile-to-brittle transition.

Our work provides a nanoscale perspective on the ductile-to-brittle transition. Previous mechanistic studies on the ductile-to-brittle transition have proposed three representative models. The Rice-Thomson model^12^ focuses on the competition between crack-tip dislocation emission and cleavage fracture in a dislocation-free crystal. This model distinguishes between completely brittle crystals that fail by cleavage fracture and ductile crystals that involve crack-tip dislocation emission. The Argon model^6^ recognizes two different ductile fracture modes in the ductile-to-brittle transition: one controlled by crack-tip dislocation emission and the other by dislocation migration. However, the understanding of the ductile-to-brittle transition in the crack-tip process zone remains limited. In contrast, the Cottrell model^42^ identifies a mixed ductile-brittle response in the crack-tip process zone, which controls the upper shelf of the ductile-to-brittle transition temperature. This response is characterized by grain-level plastic deformation and fracture at the microscale, and it can be reflected as the “one-third ductile grain condition” for semi-ductile materials like steel. Our work aligns with the Cottrell Model, emphasizing the critical role of the fracture process zone in the ductile-to-brittle transition. However, it reveals a different mixed ductile-brittle response occurring in the crack-tip process zone, which predominates at the nanoscale for less ductile materials such as BCC-refractory metals. This mixed response manifests as an alternating sequence of plastic shearing and local separation, with their competition being significantly influenced by strain rate and temperature, thereby governing the ductile-to-brittle transition. Our results underscore the crucial need to study the factors that control this key alternating sequence to fully understand the ductile-to-brittle transition in refractory transition metals.

In summary, by combining in situ atomic-resolution HRTEM experiments and MD simulations, we have revealed the atomic-scale dynamic processes of fracture in BCC Mo nanocrystals. We find that nanoscale crack growth involves the alternating processes of crack blunting accompanied by local sample thinning and crack extension along with crack sharpening. The underlying processes involve crack-tip dislocation nucleation, motion, interaction, and local separation normal to the crack plane. Our MD results show the temperature and strain rate effects on the alternating processes of crack blunting and crack extension, thereby providing insights into the dislocation-mediated mechanisms of ductile-to-brittle transition in BCC-refractory metals. Broadly, the ability to resolve the atomic-scale crack blunting and sharpening processes, which are correlated to the observed dislocation processes, allows high-resolution experimental studies to pursue a deeper understanding of the microscopic mechanisms of ductile-to-brittle transition in engineering metals and alloys.

## Methods

### In situ HRTEM experiment

TEM samples were prepared using an FEI dual-beam focused ion beam (FIB) instrument. The [001] zone axis of a bulk Mo single crystal was determined using electron backscattered diffraction. A thin slice normal to the [001] zone axis was lifted out and transferred onto a tensile loading device in the chamber of the FIB instrument. The sample was further thinned using a Ga^+^ ion beam until its thickness was reduced below 100 nm. Subsequently, the sample surface was polished at 2 kV. The tensile loading device consists of two bimetallic strips, as illustrated in Supplementary Fig. 5. Each strip comprises two metallic beams with large but different thermal expansion coefficients. The two metallic beams were bonded by hot pressing to form a bimetallic strip. The two bimetallic strips were then symmetrically fixed in parallel on a TEM copper-ring grid (Supplementary Fig. 5a). A tensile sample for in situ HRTEM imaging was placed on top of the two bimetallic strips and welded by Pt inside the FIB (Supplementary Fig. 5b). To apply tensile loading, the beams with a larger thermal expansion coefficient were placed inside. Upon heating by a Gatan double-tilt heating holder, the two strips underwent outward bending deformation, thus stretching the TEM sample (Supplementary Fig. 5c). During the in situ TEM experiments, the temperature of the sample holder was maintained below 50 °C, which is only ~0.17 of the melting point of Mo. This temperature ensures that the sample heating process has a negligible effect on dislocation activation and atom diffusion during in situ testing. Hence, the TEM results can reflect the room-temperature behavior of Mo. All in situ experiments were conducted in an FEI Titan ETEM at 300 kV.

The FIB-prepared samples have a thickness of about 80 nm, with a damage layer of about 5 nm. These samples do not have a pre-crack. During the in situ HRTEM experiment, a main crack nucleated and grew from a sample edge due to the large applied tensile strain. Supplementary Fig. 6 shows low-magnification TEM images taken before and after crack formation. As the applied tensile strain increased, the sample became thinner due to lateral contraction. In particular, the region where the crack nucleated and extended underwent larger local tensile strains, causing it to become much thinner than the rest of the sample. As a result, the thickness of the FIB-damage layer was considerably reduced in the cracked region. HRTEM images obtained at under-focus and over-focus can display diffraction contrast resulting from the thickness variation, as shown in Supplementary Fig. 7, providing evidence of reduced thickness in the cracked region. Hence, the effect of FIB-induced surface damage is likely much reduced. In addition, the observed mode of crack extension and associated dislocation activities are qualitatively consistent with our atomistic simulations. This agreement provides further support of the reduced effect of FIB-induced surface damage on our results.

To estimate the applied load on the crack during crack-tip dislocation emission, we used a representative HRTEM image to obtain local strain maps near the crack tip with a commercial package of geometric phase analysis (GPA). Then we obtained the average tensile strain and stress normal to the crack plane to estimate the stress intensity factor *K*. As described in detail in Supplementary Discussion 2, the estimated stress intensity factor is *K*. This *K* value, which drives dislocation emission and crack extension, is reasonably consistent with recent predictions by Mak et al.^24^. The difference in *K* between experiment and prediction could be attributed to the approximate nature of our estimation, as it does not account for factors such as crack blunting and deviations of the crack plane from (010), etc.

### Atomistic simulation

MD simulations were conducted to investigate crack growth in two types of single-crystal Mo samples. The first type has an in-plane size of 15.4 nm × 9 nm and a thickness of 4.2 nm (Supplementary Fig. 8a). An edge crack with a length of 4.9 nm was created by removing a layer of atoms on a horizontal (010) plane. This simulation focused on studying the processes of crack-tip blunting. The second type of sample has a size of 314.4 nm × 41.2 nm × 11.3 nm with a long (010) crack (Supplementary Fig. 8b). This sample facilitated the study of extensive crack growth. The periodic boundary condition was applied only in the [010] direction. In the MD simulations, a range of temperatures from 10 to 400 K was studied. A uniaxial tensile load was applied along the [010] direction at a constant strain rate in the range of 1 × 10^7 ^s^−1^–1 × 10^9 ^s^−1^. The time step was 1 fs. The simulations employed an EAM potential^31^ and an MEAM potential^39^ of Mo and were performed using the large-scale atomic/molecular massively parallel simulator (LAMMPS)^43^. Molecular statics calculations were also performed to investigate the crack response in the low-temperature limit close to zero K (Supplementary Discussion 1).

### Supplementary information

Supplementary Information

Peer Review File

Description of Additional Supplementary Files

Supplementary Movie 1

Supplementary Movie 2

## Data Availability

The data supporting the conclusions of this work in the main text or the Supplementary Information are available from the corresponding authors upon request.

## Code availability

Codes for molecular dynamics simulations via LAMMPS are available from the corresponding authors upon request.
