# Supplementary material for: Nanoscale ductile fracture and associated atomistic mechanisms in a body-centered cubic refractory metal
Source: Nat Commun. 2023 Sep 8;14:5540. doi: 10.1038/s41467-023-41090-3 (PMC10491606; doi:10.1038/s41467-023-41090-3)
Supplement: Supplementary file 1 — Supplementary Information [file 41467_2023_41090_MOESM1_ESM.pdf]

Supplementary Information

Nanoscale fracture and associated atomistic mechanisms in a body-centered cubic refractory metal

Yan Lu[1†], Yongchao Chen[2,3†], Yongpan Zeng[4], Yin Zhang[3], Deli Kong[1], Xueqiao Li[1], Ting Zhu[3*], Xiaoyan Li[2*], Shengcheng Mao[1], Ze Zhang[5], Lihua Wang[1*], Xiaodong Han[1*]

[1]Beijing Key Lab and Institute of Microstructure and Properties of Advanced Materials, Beijing University of Technology, Beijing 100124, China

[2]CAS Key Laboratory of Mechanical Behavior and Design of Materials, Department of Modern Mechanics, University of Science and Technology of China, Hefei 230026, China

[3]Woodruff School of Mechanical Engineering, Georgia Institute of Technology, Atlanta, Georgia, 30332, USA

[4]Centre of Advanced Mechanics and Materials, Applied Mechanics Laboratory, Department of Engineering Mechanics, Tsinghua University, Beijing 100084, China

[5]State Key Laboratory of Silicon Materials, Zhejiang University, Hangzhou, 310008, China

[†]Contribute equally to this work

*Corresponding authors: wlh@bjut.edu.cn (L.W.), xdhan@bjut.edu.cn (X.H.); xiaoyanlithu@tsinghua.edu.cn (X.L.); ting.zhu@me.gatech.edu (T.Z.)

Supplementary figures

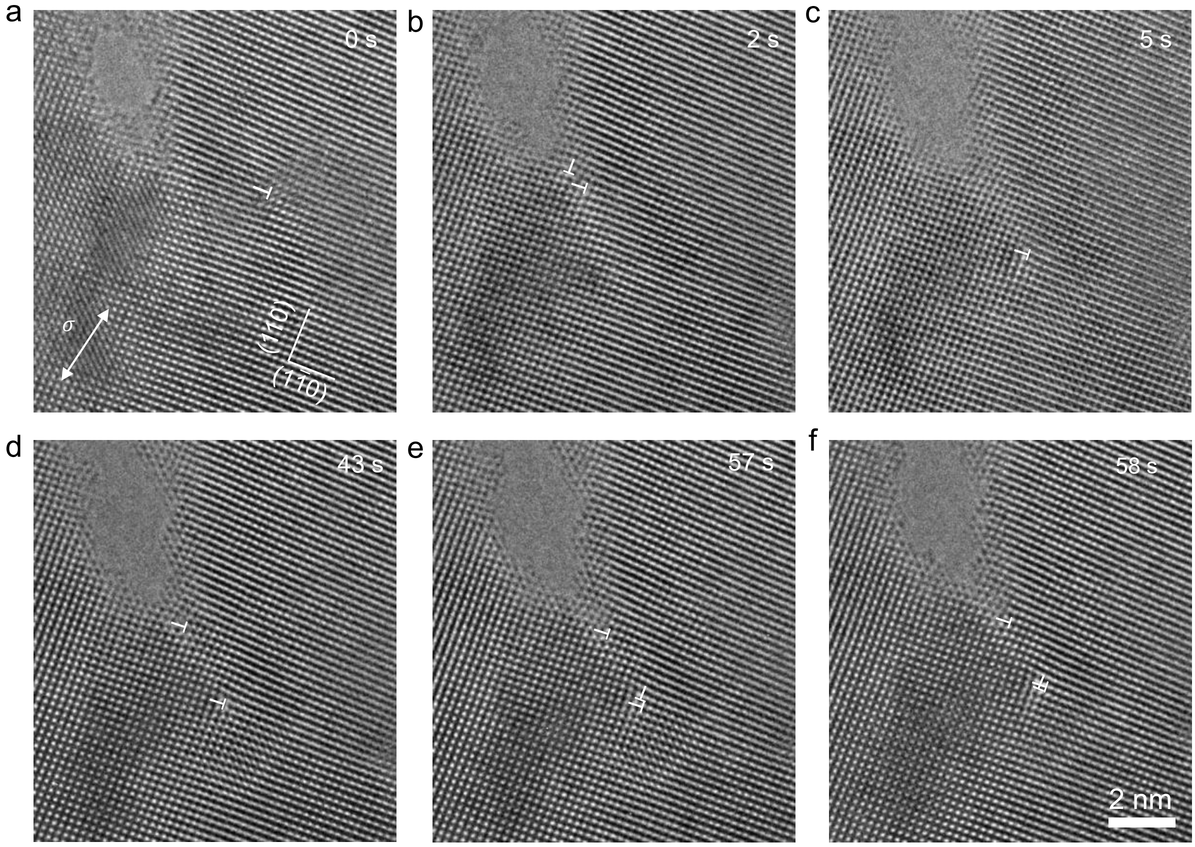

Supplementary Figure 1 Additional *in situ* HRTEM result similar to those in Figs. 1 and 2. The white arrow indicates the tensile loading direction. (a) The crack tip emits dislocations with the projected Burgers vector of 1/2 <110>{110}. (b – f) With increasing load, dislocations move away from the crack tip and more dislocations are emitted. The crack extends by ~ 4 nm within 58s.

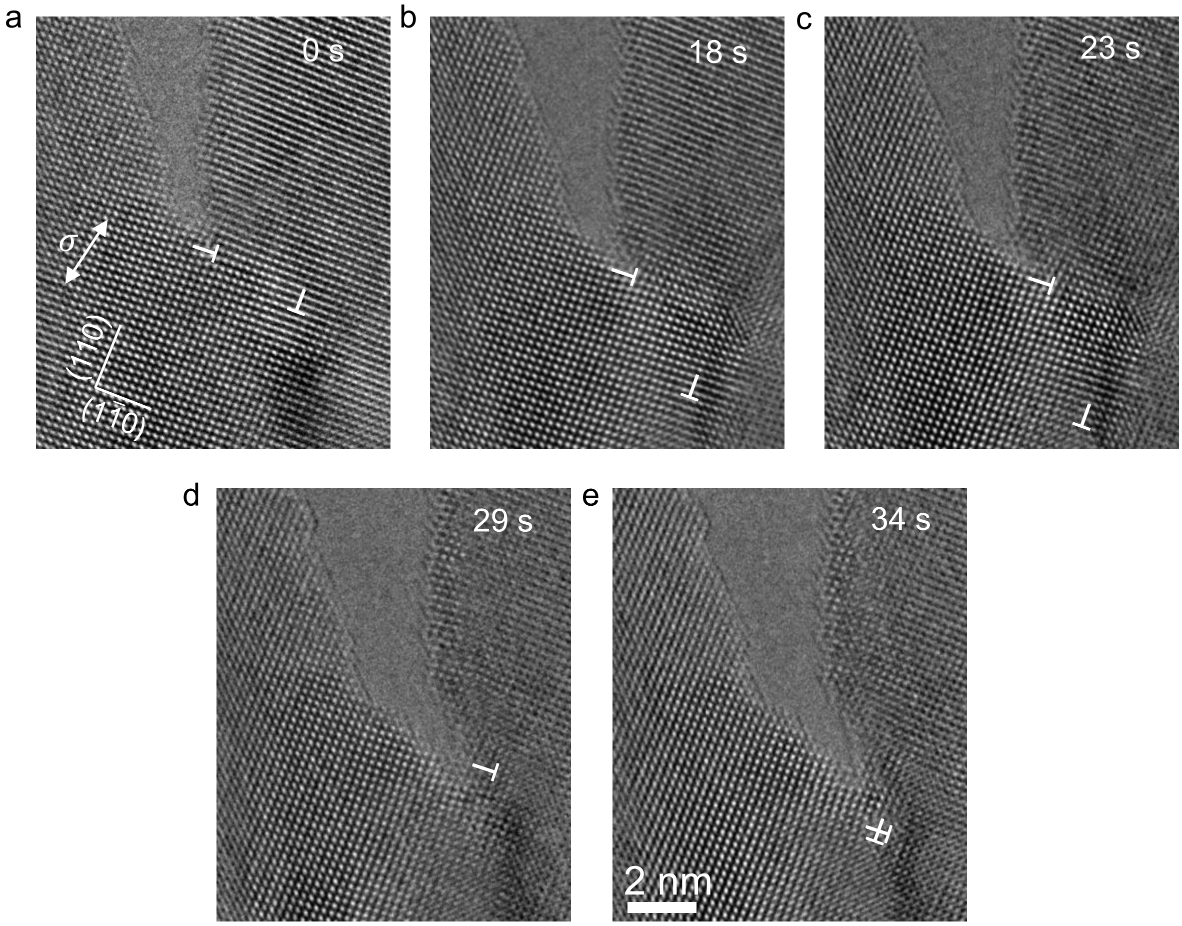

Supplementary Figure 2 Additional *in situ* HRTEM result similar to those in Figs. 1 and 2. The white arrow indicates the tensile loading direction. (a) The crack tip emits 1/2 <111>{110} dislocations with the projected Burgers vector of 1/2 <110>{110}. (b – e) With increasing load, dislocations move away from the crack tip and more dislocations are emitted. The crack extends by ~2 nm within 34s.

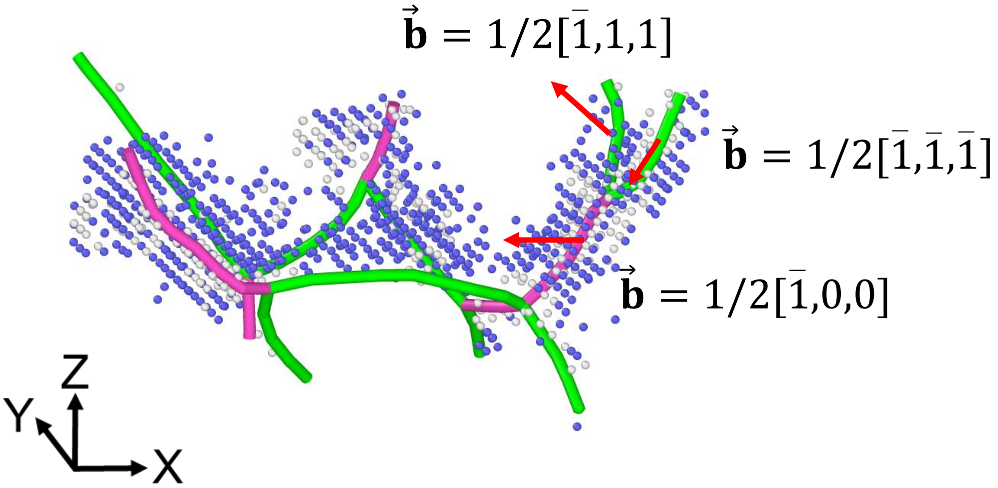

Supplementary Figure 3 MD image showing a 3D "Y"-shaped dislocation lock structure (in the right part of the MD image). It is analyzed by the visualization software OVITO.

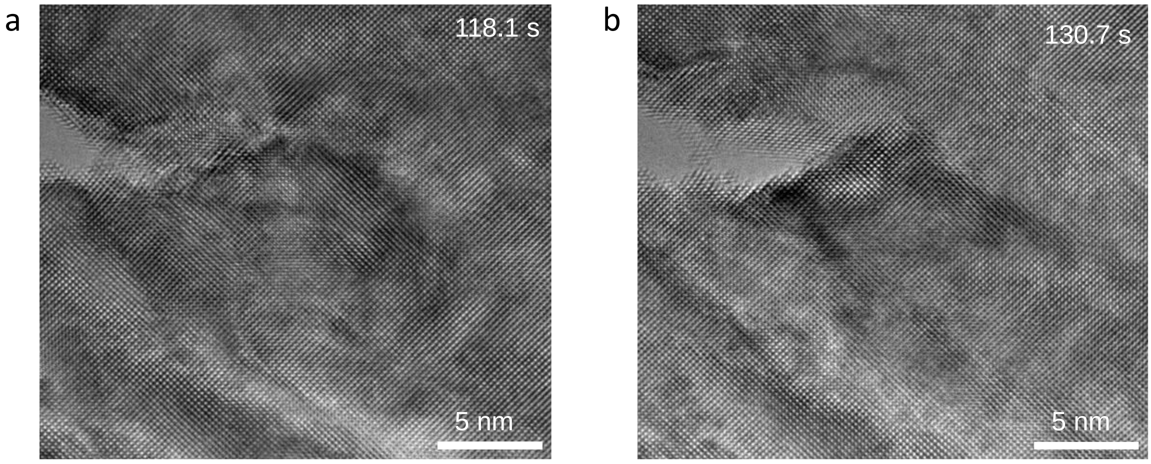

Supplementary Figure 4 *In situ* HRTEM observations of the alternating processes of plastic shearing (leading to crack blunting) and separation normal to the crack plane (resulting in crack extension). (a) A representative HRTEM image showing the blunted crack tip due to plastic shearing and local sample thinning, (b) A subsequent HRTEM image showing a stepwise increase of the crack length due to separation normal to the crack plane.

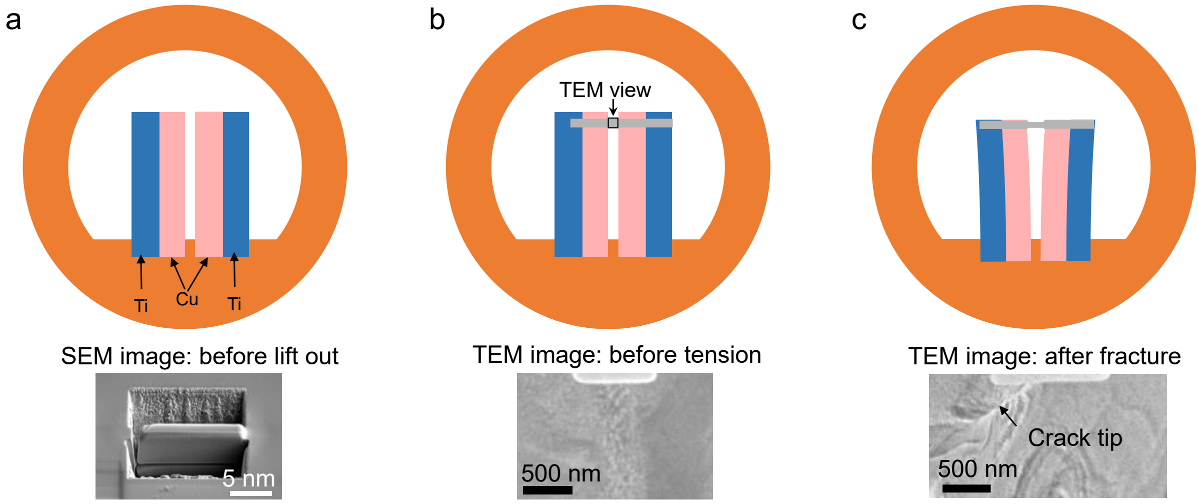

Supplementary Figure 5 Schematic illustration of the tensile device used for straining experiments near room temperature. (a) The tensile device consists of bimetallic Cu and Ti strips glued on a TEM grid. The lower SEM image shows a slice of Mo single crystal is trenched out from a bulk Mo sample before transferring to the tensile device. (b) A tensile sample for in situ HRTEM imaging is placed on top of the two bimetallic strips. The lower TEM image shows the tensile sample before loading. (c) The heated bimetallic strips produce outward bending deformation, thus stretching the TEM sample. The lower TEM image shows the formation of a crack from the edge of the tensile sample during an *in situ* straining experiment.

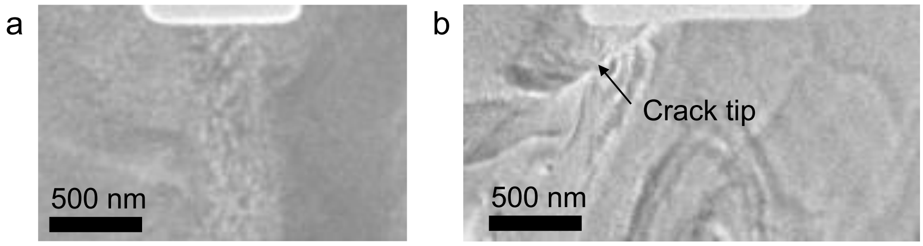

Supplementary Figure 6 Low-magnification TEM images before (a) and after (b) tension of a FIB-prepared sample. The images were acquired without applying the object lens since the beam direction is just along [001] zone axis.

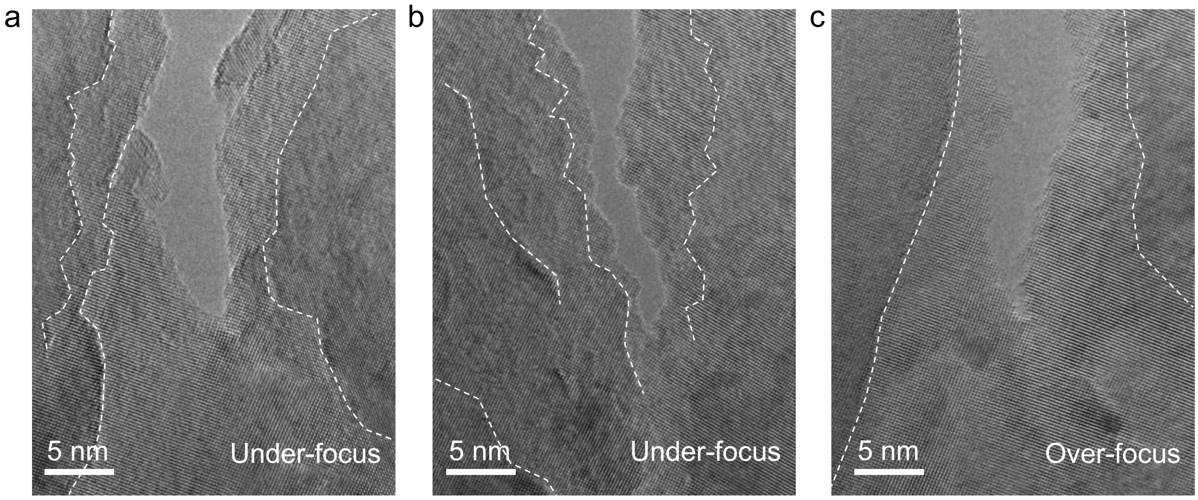

Supplementary Figure 7 Three HRTEM images including the crack tip at different focus values. (a, b) Under-focus. (c) Over-focus. The white dashed lines indicate the edges between areas with large diffraction contrast. The contrast near the crack tip is brighter, indicating a smaller thickness, which implies that the top and bottom surfaces near the crack tip are newly generated.

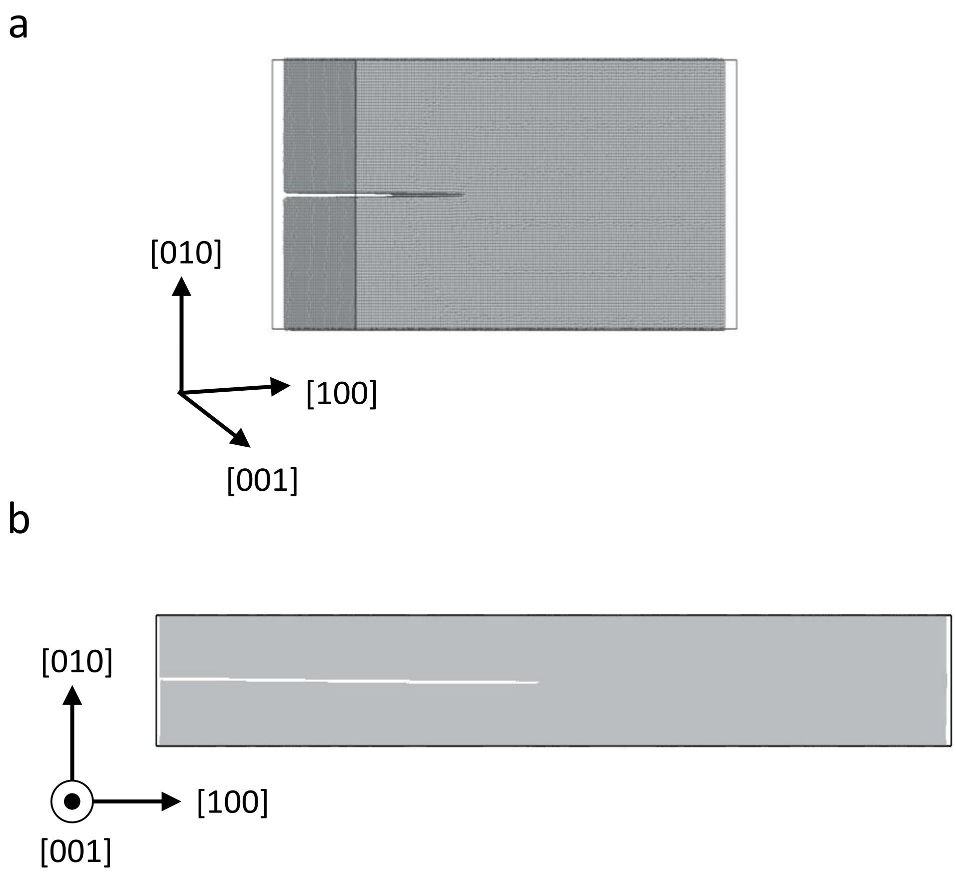

Supplementary Figure 8 MD setups showing Mo thin films with a pre-existing edge crack. (a) One setup with an in-plane size of 15.4 nm × 9 nm and a thickness of 4.2 nm. The edge crack of length 4.9 nm is created by removing a layer of atoms on a horizontal (010) plane. (b) Another setup with a long crack for investigation of extensive crack growth. The sample size is 314.4 nm × 41.2 nm × 11.3 nm.

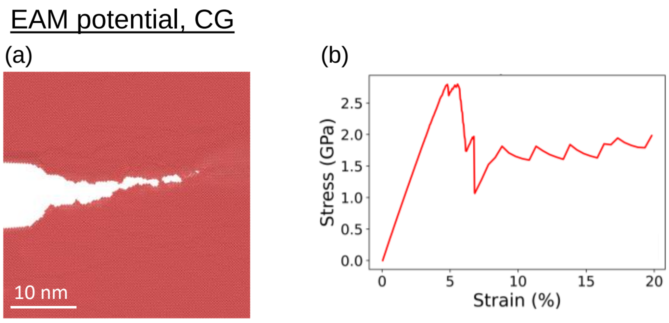

Supplementary Figure 9 MD results at the quasi-static, zero K limit. (a) An extended crack at an applied tensile strain of 17%. (b) Tensile stress-strain curve of the cracked system.

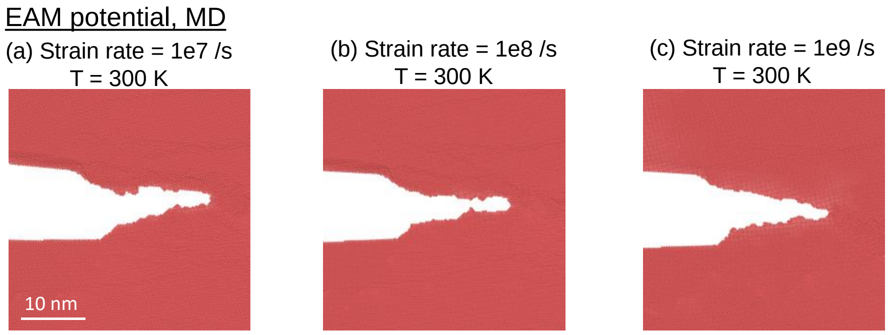

Supplementary Figure 10 MD results at a temperature of 300K and different strain rates of (a) 10^7/s, (b) 10^8/s, and (c) 10^9/s.

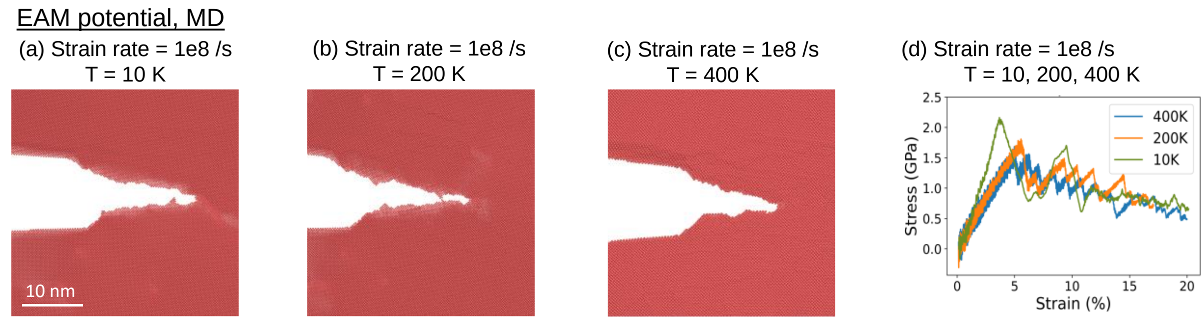

Supplementary Figure 11 MD results at a strain rate of 1e8/s, different temperatures of (a) 10 K, (b) 200 K and (c) 400 K, with the corresponding stress-strain curves in (d).

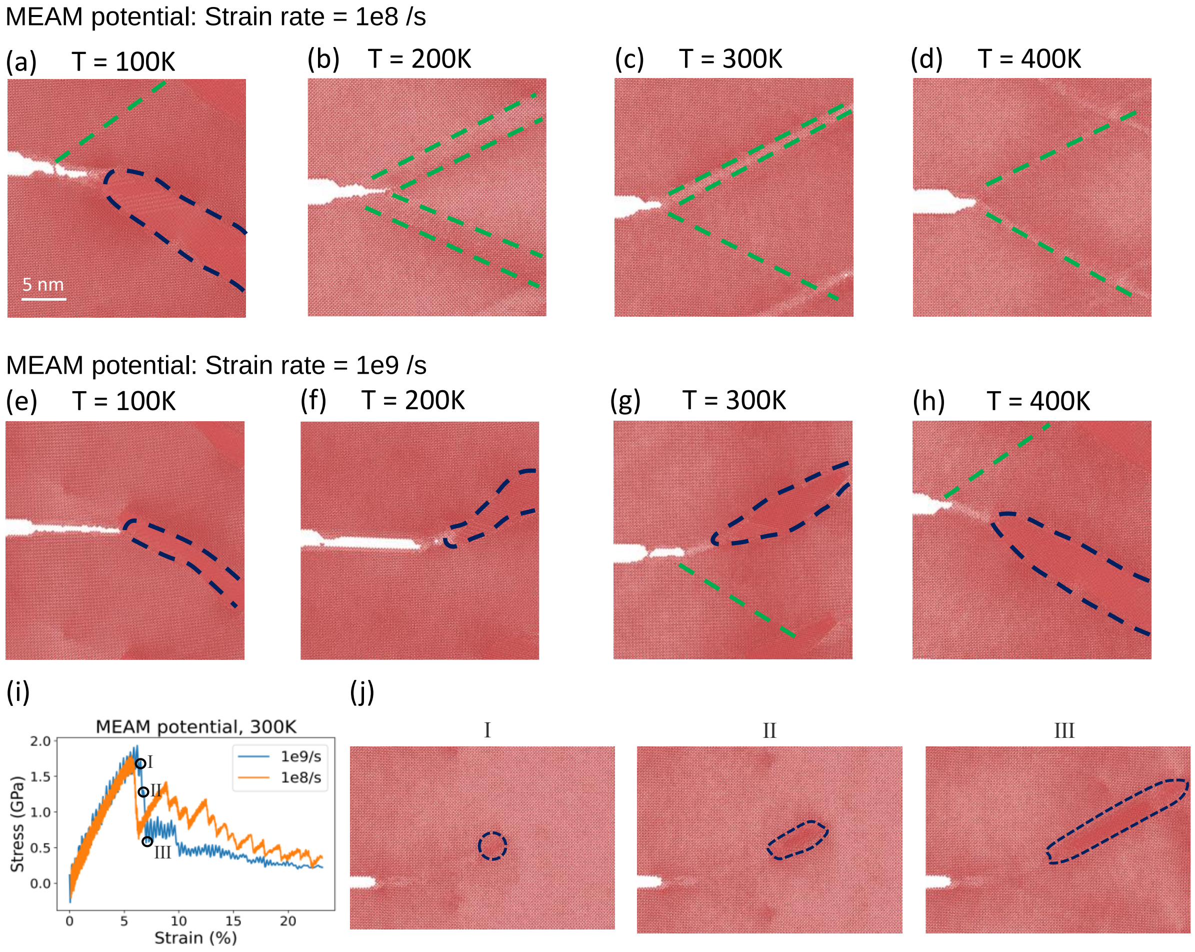

Supplementary Figure 12 MD results from an MEAM potential. (a-d) At the temperatures of 100 K, 200 K, 300 K and 400 K under the same applied strain rate of 10^8 /s, respectively. (e-h) At the temperatures of 100 K, 200 K, 300 K and 400 K under the same applied strain rate of 10^9 /s, respectively. Slip traces produced by dislocation glide are marked by green dashed lines. The deformation twin band is enclosed by dark blue dashed lines. (i) Comparison of stress-strain curves of the cracked system for the strain rates of 10^8 /s and 10^9 /s at 300 K. (j) MD snapshots showing the formation and growth of a deformation twin (enclosed by dark blue dashed lines) from a screw dislocation (I to III) at the high strain rate of 10^9 /s.

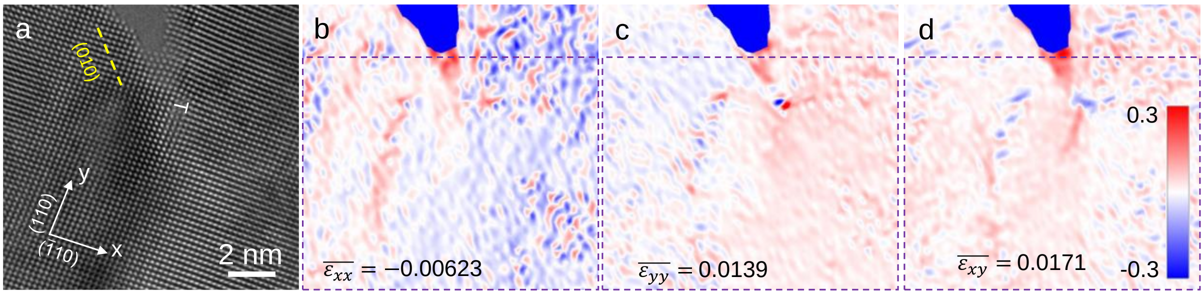

Supplementary Figure 13 HRTEM image and corresponding GPA strain mapping in front of the crack tip. (a) HRTEM image same as Fig. 1d in the main text. The (010) plane marked by yellow dashed line is the crack plane. The directions of *x* and *y* used in GPA strain mapping are aligned with [110] and $[\bar{1}10]$, respectively. The average strain components over the dashed black boxed region (b) in *x* direction is $\overline{\varepsilon_{xx}}$=−0.00623, (c) in *y* direction $\overline{\varepsilon_{yy}}$=0.0139, and (d) in *xy* direction $\overline{\varepsilon_{xy}}$=0.0171.

Supplementary Discussion 1

Atomistic simulations. In addition to the molecular dynamics (MD) results shown in Fig. 4, we have performed extra molecular statics (MS) and MD simulations at different strain rates and temperatures by first using the same EAM potential[1] discussed in the main text. As discussed in detail below, these MS and MD results show the crack growth mode by the alternating processes of plastic shearing and normal separation, which is consistent with both HRTEM observations and MD simulations discussed in the main text. The primary effect of plastic shearing is to produce crack blunting and local sample thinning, whereas that of normal separation is to extend the crack. These results demonstrate a trend of increased crack growth with more frequent normal separations and less crack blunting under the increased strain rate and/or decreased temperature. Hence, these EAM-based MS and MD results reveal a transition between ductile crack blunting and brittle crack extension mediated by strain rate and temperature.

Below we present the detailed results of additional MS and MD simulations based on the same EAM potential discussed in the main text.

1. We performed MS simulations with different incremental loading schemes and relaxation methods for examining the quasi-static crack response at zero K. When the incremental loading step is sufficiently small, we obtained qualitatively consistent MS results. For example, we selected a few layers of atoms at the upper boundary of the simulation sample, and imposed repeatedly a small incremental displacement (giving an incremental strain of 0.1%), while fixing a few layers of atoms at the lower boundary. After each loading step, the system was relaxed using the conjugate gradient method for energy minimization. Fig. S9a shows a representative atomic configuration containing an extended crack at an applied tensile strain of 17%, and Fig. S9b shows the simulated tensile stress-strain curve of the cracked system. This MS result gives a similar crack geometry with a larger amount of crack extension and a sharper crack tip compared to the MD results at the same applied strain under different temperatures and strain rates (below). Note that the crack geometry from MS simulations also results from the same kind of growth mode of alternating plastic shearing and normal separation as MD simulations. However, thermally activated dislocation nucleation and migration are suppressed in MS simulations at zero K, thereby reducing crack-tip plastic shearing and associated crack blunting. As a result, more frequent normal separations occur along the crack plane, leading to a more brittle response at the quasi-static, zero K limit.

2. We performed MD simulations under different applied strain rates of 10^7, 10^8, 10^9 /s at the same temperature of 300 K, respectively. Figures S10a-c show a representative atomic configuration containing an extended crack under an applied tensile strain of 17% for each strain rate applied. It is seen that the resultant crack geometries are similar, but a higher strain rate leads to a larger crack extension and a sharper crack tip. These similar crack geometries result from the same growth mode by the alternating processes of plastic shearing and normal separation. However, the increased strain rate suppresses stress relaxation at the crack tip, leading to a more brittle response of enhanced crack extension and reduced crack blunting.
3. We also performed MD simulations at different temperatures ranging from 200 K to 400 K and under the same applied strain rate of 10^8 /s. Figures S11a-c show a representative atomic configuration containing an extended crack under an applied tensile strain of 17% for the temperature of 10 K, 200 K, 400 K, respectively, and Fig. S11d shows the corresponding stress-strain curves. It is seen that the resultant crack geometries are similar, but a lower temperature leads to a larger crack extension and a sharper crack tip. These similar crack geometries result from the same growth mode by the alternating processes of plastic shearing and normal separation. However, the decreased temperature suppresses stress relaxation at the crack tip, leading to a more brittle response of enhanced crack extension and reduced crack blunting.

To summarize, the above MS and MD simulations demonstrate a trend of increased crack growth with more frequent normal separation and less crack blunting under the increased strain rate and/or decreased temperature. These EAM-based results reveal a transition between ductile crack blunting and brittle crack extension mediated by strain rate and temperature.

Moreover, we have performed MD simulations using a modified EAM (MEAM) potential[2] discussed in the main text, which can capture the effect of directional bonding in BCC metals. As discussed in detail below, the MEAM-based MD results not only show the crack growth mode by the alternating processes of plastic shearing and normal separation mediated by temperature and strain rate, but also reveal a competing mode of brittle fracture through deformation twinning-induced crack extension that predominates at low temperatures and high strain rates.

First, we examine the MEAM-based MD results at different temperatures of 100 K, 200 K, 300 K and 400 K under the same applied strain rate of 10^8 /s. Figures S12a-d show a representative atomic configuration containing an extended crack under an applied tensile strain of 13% for each temperature. It is seen from Supplementary Figs. 12b-d that the resultant crack geometries

are similar, due to the same growth mode by the alternating processes of plastic shearing and normal separation. However, the decreased temperature suppresses stress relaxation at the crack tip, leading to a more brittle response of enhanced crack extension and reduced crack blunting. Moreover, Fig. S12a reveals a competing brittle fracture mode by crack extension induced by a deformation twin band (area enclosed by dark blue dashed lines) at a low temperature of 100 K. Figures S12i and j show the formation of a deformation twin band from a screw dislocation at a high strain rate and/or low temperature. As the growing twin band approaches the crack, crack-tip dislocation emission becomes more difficult, leading to brittle fracture.

Next, we examine the MEAM-based MD results at different temperatures of 100 K, 200 K, 300 K and 400 K under the same applied strain rate of 10^9 /s. Figures S12e-h show a representative atomic configuration containing an extended crack at an applied tensile strain of 13% for each temperature. It is seen that the brittle fracture mode of deformation twinning-induced crack extension predominates at the high strain rate of 10^9 /s for the temperate range studied. Nonetheless, the increased temperatures of 300 K and 400 K can activate dislocation slip (with slip traces marked by green lines), increase crack blunting, and slow down crack growth. In Fig. S12i, the stress-strain curves of the cracked system are compared for the strain rates of 10^8 /s and 10^9 /s at 300 K. It is seen that an increased strain rate leads to a higher maximum stress and a lower stress plateau, due to the occurrence of a more brittle fracture mode of deformation twinning-induced crack extension.

To summarize, the MEAM-based MD results reveal a broader range of competing processes of ductile to brittle transition in BCC crystals compared to the EAM-based MD results. The transition to deformation twinning-induced brittle fracture at low temperatures has been reported by previous experiments for bulk BCC crystals[3,4]. The corresponding in situ HRTEM experimental results are not available yet. As discussed earlier, these atomistic results would motivate further experiments with controlled temperature and strain rate to pursue a deeper understanding of ductile to brittle transition in the future.

Supplementary Discussion 2

To estimate the applied load to the crack during crack-tip dislocation emission, we used a representative HRTEM image to obtain its local strain maps near the crack tip with a commercial package of geometric phase analysis (GPA). Then we obtained the average tensile strain and stress normal to the crack plane for an estimate of stress intensity factor *K*. More

specifically, Supplementary Figure 13 shows a representative HRTEM image and corresponding GPA strain maps of ε_{xx}, ε_{yy} and ε_{xy}. Note that the x and y directions (marked in Fig. S13a) are aligned with [110] and $[\bar{1}10]$, respectively, and the overall crack plane is approximately along the (010) plane (marked by a yellow dashed line in Fig. S13a). From the GPA strain maps for ε_{xx}, ε_{yy} and ε_{xy}, we calculated the average strain components of $\overline{\varepsilon_{xx}}$, $\overline{\varepsilon_{yy}}$ and $\overline{\varepsilon_{zz}}$ over the dashed line boxed region in Fig. S13b-d, respectively. The numerical results of $\overline{\varepsilon_{xx}}$, $\overline{\varepsilon_{yy}}$ and $\overline{\varepsilon_{zz}}$ are listed in Fig. S13b-d, respectively. Then we used the strain transformation equation to calculate the average tensile strain normal to the crack plane (010) (with a rotation of 45° from the (110) plane) as 0.021, and further estimated the corresponding average tensile stress = 7 GPa (using the Young's modulus of Mo of 335.3 GPa). Taking a typical crack length of a = 500 nm in our experiment, we estimated the stress intensity factor $K = \sigma\sqrt{\pi a}$ as 8.8 MPa$\sqrt{\text{m}}$. This estimated K value for driving dislocation emission and crack extension is reasonably consistent with recent predictions (e.g., ~2.5MPa$\sqrt{\text{m}}$ for dislocation emission at a 100/110 crack in Mo) by Mak et al.[5]. The difference in K between experiment and prediction could be attributed to the approximate nature of our estimate without accounting for the effects of crack blunting and crack plane deviation from (010), etc.

Supplementary References

1 Ackland, G. J. & Thetford, R. An improved N-body semi-empirical model for body-centred cubic transition metals. *Philosophical Magazine A* **56**, 15-30 (1987).

2 Park, H. *et al.* Ab initio based empirical potential used to study the mechanical properties of molybdenum. *Physical Review B* **85**, 214121 (2012).

3 Cottrell, A. H. The Bakerian Lecture, 1963. Fracture. *Proceedings of the Royal Society of London A* **276**, 1-18 (1963).

4 Cottrell, A. H. & Rotherham, L. Mechanics of fracture in large structures. *Proceedings of the Royal Society of London A* **285**, 10-21 (1965).

5 Mak, E., Yin, B. & Curtin, W. A. A ductility criterion for bcc high entropy alloys. *Journal of the Mechanics and Physics of Solids* **152**, 104389 (2021).
