# Supplementary material for: Nanoscale ductile fracture and associated atomistic mechanisms in a body-centered cubic refractory metal
Source: Nat Commun. 2023 Sep 8;14:5540. doi: 10.1038/s41467-023-41090-3 (PMC10491606; doi:10.1038/s41467-023-41090-3)
Supplement: Supplementary file 3 — Description of Additional Supplementary Files [file 41467_2023_41090_MOESM3_ESM.pdf]

Description of Additional Supplementary Files

File Name: Supplementary Movie 1
Description: Crack propagation and dislocation movement at atomic scale.

File Name: Supplementary Movie 2
Description: Crack propagation and dislocation movement at low magnification.
